# From animal models to gut-on-chip: the challenging journey to capture inter-individual variability in chronic digestive disorders

**DOI:** 10.1080/19490976.2024.2333434

**Published:** 2024-03-27

**Authors:** Aicha Kriaa, Vincent Mariaule, Charlotte De Rudder, Amin Jablaoui, Harry Sokol, Paul Wilmes, Emmanuelle Maguin, Moez Rhimi

**Affiliations:** a Université Paris-Saclay, INRAE, AgroParisTech, Micalis Institute, Jouy-en-Josas, France; b Luxembourg Centre for Systems Biomedicine, University of Luxembourg, Esch-sur-Alzette, Luxembourg; c INSERM UMRS-938, Centre de Recherche Saint-Antoine, CRSA, AP-HP, Sorbonne Université, Paris, France; d Department of Life Sciences and Medicine, Faculty of Science, Technology and Medicine, University of Luxembourg, Esch-sur-Alzette, Luxembourg

**Keywords:** Gut microbiome, host response, tools, microfluidic, gut-on-chip, animal models, variability, holobiont, chronic digestive diseases

## Abstract

Chronic digestive disorders are of increasing incidence worldwide with expensive treatments and no available cure. Available therapeutic schemes mainly rely on symptom relief, with large degrees of variability in patients’ response to such treatments, underlining the need for new therapeutic strategies. There are strong indications that the gut microbiota’s contribution seems to be a key modulator of disease activity and patients’ treatment responses. Hence, efforts have been devoted to understanding host–microbe interactions and the mechanisms underpinning such variability. Animal models, being the gold standard, provide valuable mechanistic insights into host–microbe interactions. However, they are not exempt from limitations prompting the development of alternative methods. Emerging microfluidic technologies and gut-on-chip models were shown to mirror the main features of gut physiology and disease state, reflect microbiota modification, and include functional readouts for studying host responses. In this commentary, we discuss the relevance of animal models in understanding host–microbe interactions and how gut-on-chip technology holds promises for addressing patient variability in responses to chronic digestive disease treatment.

## Introduction

Chronic non-communicable diseases are lifelong-debilitating conditions with increasing incidence worldwide. They remain the leading cause of death worldwide representing around 74% of all deaths globally according to the World Health Organization.^1^ The socioeconomic burden of non-communicable diseases is daunting, with a predicted cost of $47 trillion over the next 20 years.^2^ Of these conditions, Inflammatory Bowel Diseases (IBD), including Crohn’s disease (CD) and ulcerative colitis (UC), affect about 6.8 million people worldwide.^3^ Irritable bowel syndrome (IBS), a common disorder of gut–brain interactions, is estimated to affect about 1 in 10 people worldwide, with significant societal and economic repercussions.^4^ The pathophysiology of IBD and IBS is intricate and the role of risk factors including the genetic background, lifestyle, diet, and gut microbiota is believed to vary across the globe. New therapeutics are increasingly sought to address both conditions, and significant efforts have been undertaken, yet, no cure is at hand today. Despite proven benefits, treatment schemes for IBS and IBD entail significant drawbacks, ranging from adverse side effects and primary non-response, to the loss of secondary response.^5,6^

## Exploring the role of the microbiome in chronic digestive disorders: Current model systems and their limitations

### The role of the microbiome

Several reports now point to the role of gut microbes in influencing response to treatments and potentially shaping patients’ response to therapies.^7^ As gut microbes are believed to modulate drug oral bioavailability through the production of microbial drug-metabolizing enzymes, immunomodulatory properties,^8,9^ or via microbiota-host cometabolism,^7,9^ they can influence drug pharmacodynamics and pharmacokinetic mechanisms alongside patients’ responses to treatment. Inter-individual variability in gut microbiota composition may in particular contribute toward individual variability in responses to a given therapeutic intervention.

### Current models

**Figure 1.**
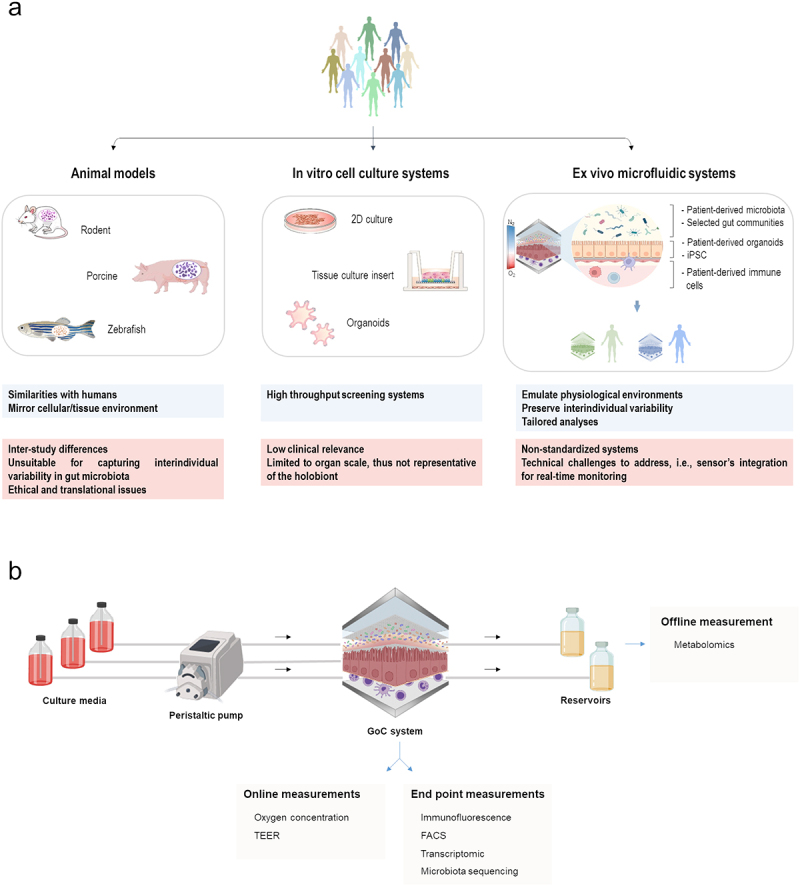
GoC systems in research (a) Overview of the pros and cons of commonly used models in microbiome research. Main advantages of each system are emphasized in blue, while drawbacks are shown in red. (b) Examples of features and design for GoC systems.

As available models rarely reflect such variability, and considering the variety of confounding factors, predicting the response of the host and its gut microbiota to a given treatment, and therefore disease management, remains highly challenging. Furthermore, much of our current mechanistic knowledge of these diseases has so far relied on *in vivo* models, which are often criticized for their relatively poor ability to predict disease outcomes and clinical efficacy in humans, and the application of which is limited by ethical considerations (Figure 1(a)). We are yet to reach a comprehensive understanding of IBD and IBS pathogenesis, which will probablycall for a ‘holobiont’ approach targeting the host and its microbiomes. There is no doubt that animal models have thus far been the cornerstone of research on elucidating the mechanisms underlying host-microbe crosstalk in many disease states, including in IBD and IBS.^10^ Indeed, the use of various species ranging from invertebrates, and non-mammalian vertebrates to mammals (*e.g*. mice, rats, pigs) during the late twentieth and early twenty-first centuries has been successful in many areas, notably by improving the current knowledge of cellular signaling pathways, identifying key targets for treatment, and guiding the design of promising therapeutic approaches in both conditions.^11–14^ Relevant examples include, but are not limited to gnotobiotic humanized models with a single gut microbe, complex or minimal consortia as well as transplantations of entire gut microbial communities from specific human donors. While showing merits and limitations, each of these approaches aimed to explore host-microbe interactions, particularly immune-microbe feedbacks, to infer causal relationships and to assess new therapeutics. The field of IBD research, for instance, has been largely based on murine studies to better understand colitis and explore the role of a variety of factors in shaping host response and disease susceptibility. As such, evidence of colitis induction was reported for specific microbial communities or species including *Helicobacter* spp. and *Enterobacteriaceae*.^15,16^ Along these lines, monocolonization studies have shown significant improvements with select species of *Clostridiales* and *Bacteroides*, which were shown to trigger the activation of colonic regulatory T cells (Treg) and consequently counteract mucosal inflammation.^17^ These findings will bring us one step closer to defining potential microbial culprits behind the disease in view of developing next generation probiotics. Yet, one has to bear in mind that colonization with either single gut immunomodulatory strains or different complex microbiota may elicit varying responses and establish diverse gut immune landscapes. Individual microbes may exhibit contrasting behaviors when studied in a multispecies community, as many metabolites, due to the inherent emergent properties of the ecosystem, are only produced in the presence of other bacteria.^18^ The gut ecosystem is highly complex featuring cooperative and competitive interactions between intestinal microbes. Recapitulating these polymicrobial interactions with the host requires embracing a holistic ‘holobiont’ view of host-microbe interactions. In this regard, it has been demonstrated that transplantation of human fecal microbiota from donors with IBD can induce intestinal inflammation in susceptible mice.^19^ Such changes were independent of diet or body weight and were driven by alterations in gut microbiota composition, thereby stressing the key role of resident microbes in the disease. Nevertheless, the limited insights into the subtle intricacies of the gut microbiota and the lack of standardized protocols for such approaches pose key constraints to the effective translation of results obtained through these models. Major limitations to the current models are that they commonly fail to capture all features and functions of human donors’ microbiomes, and can themselves be sources of response variability (Figure 1(a)). The latter could be related to, but is not restricted to the genetic background. The gut microbiome contains approximately a hundredfold more genes than its human host,^20,21^ and has a variety of metabolic capacities (*e.g.*, vitamin synthesis, fiber degradation, de- and reactivation of drugs) that humans lack or have limited ability to perform. The gut microbiota thus stands out as a source of functional diversity that can modulate an individual’s response and differentially predispose humans to disease.^22^ These findings highlight the limitations of the ‘one-stool fits all’ approach to managing diseases and stress the current challenge of addressing gut microbiome functional diversity in most scientific studies. This question is not new to science and is often magnified by the multifactorial nature of most explored diseases and the existence of varied confounding factors that presumably underlie inconsistencies among studies addressing the contribution of gut microbes to pathogenesis. Each individual is home to an idiosyncratic set of gut species and strains, and while most models share common microbial features with humans, they do not represent interindividual variability. Also, experimental designs are far from being standardized as are the guidelines for human-microbiome association models aimed at inferring causal relationships. The divergence of lab mouse models among commercial suppliers and research institutes is a major driver of irreproducibility and conflicting data in basic and preclinical research. Different animal models are nowadays available for the same disease, which raises the question of whether and which model would make a significant contribution to understanding the clinical condition. However, major challenges still exist equally regarding the intervention time commonly needed to evoke physiological changes, as well as the duration before those changes would wane. To prevent overstating and ensure reproducibility, scientifically supported, common standard operating practices or guidelines is warranted. They need to be robust and flexible, leaving provision for adaptation to the rapid advances in technology. To counter the translational and ethical challenges related to animal models, more physiologically relevant culture systems that mimic key tissue features in humans are being implemented.

## Advances in gut-on-chip systems and complementarity with animal models

Microfluidic technology allows three-dimensional analysis of the morphology of intestinal epithelium without disturbing the system as well as real-time in situ monitoring of tissue function and viability. By adjusting parameters such as shear forces, oxygen concentrations, and cell types, Gut-on-chip (GoC) devices offer a high degree of flexibility that *in vivo* models cannot provide. The ability to study host–microbe interactions and diet/drug-host-microbiome using (personalized) human cells and microbiota means a step forward for the translation of preclinical findings. GoC systems appear complementary to the animal models as they provide direct *in situ* analysis that is limited in standard *in vivo* systems. In contrast, animal models provide a complex physiological environment and allow studies over several months as in chronic intestinal inflammation context. Animal models allow more in-depth study of microbiome-immune interactions, as well as of systemic effects. Furthermore, *in vivo* models allow the study of organ–organ interactions comprising the entire body, whereas organ-organ-chip, or body-on-chip technology is still in its infancy. Overall, GoC technology is a new tool that complements animal models, bringing us closer to a human model for studying host–microbiota interactions.

As we are stepping into a new era of cutting-edge technologies, microfluidic devices have been on track to becoming largely accepted as human-specific experimental platforms for preclinical research and therapeutic trials. GoC is a scientific and technological breakthrough in which biology is teamed with microtechnology to emulate key facets of human physio/pathology. Of interest, in 2018, the microfluidics market was valued at $3.6–5.7 billion,^23^ and is expected to keep growing, among others due to the upcoming changes in the regulatory frameworks for preclinical testing (FDA Modernization Act 2.0, Directive 2010/63/EU on the protection of animals used for scientific purposes), which encourage the use of alternative technologies, *e.g.*, organ-on-chips, in line with the three Rs (Replacement, Reduction, and Refinement) principle to reduce animal testing. Organ-on-chips (OoC) and GoC technology are a relatively recent addition to the landscape of *in vitro* models available for preclinical research.^24,25^ The technology emerged as a next step after simple cell culture models in the late 1900’s and the early 2000’s,^26,27^ enabling the culture of one or more different cell types in emergent, single-channel organ-on-chip devices. The field started growing exponentially after the publication of the seminal lung-on-chip paper in 2010,^28^ which presented a perfused microfluidic device, containing epithelial and endothelial cells, as well as “breathing” movements, which mimicked additional mechanical cues experienced *in vivo* and necessary for differentiation. Since then, the field has taken great strides forward following both advances in engineering (3D printing, sensor integration, scaffolding, extracellular matrix and hydrogel development, microfabrication, and introduction of oxygen gradients and mechanical stresses) and biological (combination of different cell types, induced pluripotent stem cells, organoid differentiation and culture, common or universal cell culture media for co-cultures),^29^ which has improved cytoarchitectural and cell chamber structures, as well as (3D) tissue-like differentiation of the cells. Furthermore, there is an increase in the development of multi-organ-on-chip models, making it possible to study the interaction of multiple organs, as well as off-target and systemic effects. For reviews on this topic, the reader is referred to Low et al. 2021, Leung et al. 2022 and Ingber 2022.^25,26,30^

Unsurprisingly, a wide variety of microfluidic systems and disease models based on organs-on-a-chip is emerging to take over the commercial arena.^31^ Many systems are now available either commercially or as prototypes from academic institutions that offer several readouts of cell-, tissue-, and organ-level behaviors.^32^ GoC systems are microfluidic, bioengineered, and 3D models of (regions of) the intestine. They typically consist of one or more culture chambers containing intestinal cells (*e.g.*, immune, epithelial, and endothelial), separated by micro- or nanoporous membranes, which can simultaneously act as culture scaffolds and are perfused with cell culture medium (Figure 1(b)). The microscale and presence of scaffolding and relevant 3D structures allows research of cell interactions on a physiologically representative scale. GoC allows a more physiologically relevant culture and differentiation of different intestinal cell types compared to static (tissue culture insert-based) models. GoC systems support the culture of different human cell types such as Caco-2 cells, a well-known immortalized cell line, and intestinal organoids. While Caco-2 cells harbor key features of intestinal epithelium (e.g., brush border, tight junctions, villi formation, and permeability), they fail to recapitulate normal intestinal physiology.^33,34^ On the contrary, intestinal organoids can be obtained from different intestine regions and contain differentiated cell types, thus recapitulating *in vivo* intestinal tissue architecture and multiple-cell type heterogeneity and interaction.^35^

Microfluidic GoC devices mimic the tissue-specific microenvironment by including vascular perfusion and physiologically relevant mechanic stimuli and can recapitulate tissue–tissue interaction and interaction with circulating immune cells. Once inoculated with human cells and gut microbes or communities, GoC offers the potential to mimic the complex structures and physiological functions of the human gut, both alone and when fluidically coupled together with other organ-on-chip devices to create human body-on-a-chip systems. Relevant simulated intestinal features comprise barrier function (2D cell cultures, 3D microstructures (*e.g*., villi)), and emulation of biomechanical signals (*e.g.*, shear stress and oxygen gradient) using perfused chambers. Fluids are readily controlled at the microscale to maintain co-cultures of host epithelial cells with gut microbes and preserve subtle balances of chemicals and metabolites.^36^ With the greater need for real-time data that can be used for decision-making, more emphasis has been placed on integrating biosensors within these devices. Recent advances in nano- and micro-technology have extended the types of sensors available for in-chip biological processes in so-called lab-on-chips, as well as the 3D structures of the channels. Functional readouts to assess barrier integrity,^37,38^ oxygen concentrations, and inflammatory responses (*e.g.*, cytokine profiles and short-chain fatty acid levels) can be included using on-chip biosensors, or by post-analysis of manually collected samples.

In the past ten years, a range of GoC systems have been developed, using different approaches to mimic host and microbial aspects, with different levels of personalization. Examples comprise (personalized) modeling of inflammatory processes and preclinical drug assessment in GoC devices using iPSC-derived human intestinal organoids or biopsy-derived organoids.^39,40^ Human iPSCs are good candidates to make fully personalized GoC models feasible, as they allow many cell types to be derived from the same genetic background. The improved emulation of *in vivo* physiology in GoC facilitates a more representative manner of studying host–microbe interactions in IBD and IBS via the follow-up of immune responses, such as recruitment of immune cells and neuronal-immune interaction upon stimulation with selected bacterial compounds.^41–43^ IBS and IBD phenotypes or potential therapies can be studied via co-culture with probiotic or pathogenic microorganisms, *e.g., Faecalibacterium duncaniae (previously named F. prausnitzii)*, which can exert anti-inflammatory effects through butyrate production,^44^ or with adherent invasive *E. coli*, *Shigella*, or *Candida* species which are associated with flare-ups.^42,45^ Next to this, exposure to disease-specific cocktails of pro-inflammatory cytokines or to chemical compounds, such as dextran sodium sulfate can be used,^46^ resulting in characteristic epithelial injuries and can aid in unraveling disease mechanisms. GoC allows for infection studies with live microorganisms, both thanks to perfusion of the system, as well as peristaltic-like mechanical deformations which can reduce bacterial overgrowth and allow for longer co-culture times,^47^ but can enhance colonization by other bacterial species, such as *Shigella* sp. present in IBD flare-ups.^42^ GoC devices have also demonstrated high adaptability and feasibility to co-culture human cells with – multi-species mixtures or complex human gut communities, thus allowing glimpses into personalized host–microbiota interactions (Figure 1(a)).^36,48^ As such models evolve, new avenues for better understanding disease processes are emerging. Pinpointing the exact role of relevant microbes and/or their molecules is often hampered by the inter-individual variability of microbial communities and response to select interventions. Hence, introducing a patient’s gut microbiota alongside human cells into such a system would allow the exploration of personalized responses. This would help design tailored interventions to improve patients’ health without omitting patient-specific microbial data in clinical practice. While human epithelial cell culture is performed under aerobic conditions, the gut microbiome requires anaerobic conditions. The integration of an oxygen gradient across the different culture chambers of a GoC represents an essential improvement in mimicking the gut’s physiological condition.^36,48,49^ The presence of an oxygen gradient has been shown to have an impact on the diversity, richness, and spatial distribution of bacteria but also in gene expression patterns of human epithelial cell line.^36,48,49^ This provides a further step to recapitulating physiologically relevant host–microbiota interactions using microfluidic technology (Figure 1).

## Limitations and required improvements in the application of GoC for preclinical research into chronic digestive disorders

GoC systems constitute promising tools of which technological development is still in its infancy that will benefit from future methodological and technological advances. The commercialization of current GoC systems and the development of automated control systems (e.g., Zoë-CM2™ Culture Module from Emulate and Omi platform from Fluigent) and chips incorporating pre-qualified cells (to develop *in vitro* cell cultures having the same ratio of epithelial cell subtypes and transcriptome profile as human tissue; *e.g*., biopsy-derived primary human organoids) have paved the way toward the definition of validated methods and performance criteria, ensuring the GoC reliability, robustness, and consistency. However, due to the instrument investment requirements (*i.e.*, the “lab around the chip”) and the technical challenges associated with fabricating, setting up, and operating even simple GoC systems, the technology is not widely available to the host-microbiome research community yet. In-house fabrication of GoC and their operation remain sources of variability. High throughput experiments using GoC are limited by their compatibility with standard laboratory equipment, the number and type of pumps available, and the current lack of standardization, which requires testing and optimization of assays. While multiple sensors can be integrated, many devices rely largely on endpoint measurements, limiting real-time monitoring. GoC fails to fully reflect the complexity of *in vivo* organs, both on a 3D engineering level, as in the number of interactions they can mimic. GoC systems typically mimic the colon or small intestinal sections of the human intestinal tract, but fail to recapitulate the different physiological conditions, microbiome compositions, and densities occurring over the length of the intestinal tract, making it currently impossible to study these in a single GoC.^24^ At the gut level, the diversity of human cells (epithelial, endothelial, immune cells, and fibroblasts) and of the microbiome (bacteria, fungi, and viruses) represent essential factors which shape gut physiology. Importantly, the culture of human microbial communities, *e.g.*, fecal communities, *in vitro*, is known to induce shifts in the composition of said communities, often via overgrowth of fast-growing, or facultative anaerobic species, rendering it difficult to mimic actual *in vivo* interactions. GoC devices have been shown to allow the co-culture of a single bacterial strain or a consortium of synthetic bacteria with human cells for up to 96 hours.^36,50^ With the integration of oxygen gradient, the culture of more complex microbial communities has been made possible with the recent example of the intestine-on-a-chip device, which allowed the co-culture of a microbiota isolated from fresh infant stool and primary human intestinal cells for five days.^48^ Moreover, provided further advances in co-culturing, GoC can be leveraged in a culturomics approach through the culture of complex gut-derived communities to identify novel species and strains involved in pathogenesis, or with potential for disease mitigation.^51,52^ This is particularly important given the limited mechanistic understanding of the etiology of these diseases. Despite these recent progress, the long-term culture of a stable complex human microbiome represents one of the main challenges for GoC technology. Sensors may represent a first approach to overcome such limitation. The combination of sensors for microenvironment parameters (*e.g*., pH, oxygen, temperature, ions, cytokine, and metabolites) could be used to perform a dynamic survey of culture conditions in response to chemical or bacterial alteration which might help to sustain complex microbiome viability and stability.

Several hurdles need to be overcome to ensure wide integration of GoC for host–microbe interaction research in the context of chronic digestive disorders. Standardization of device fabrication and operation will partly remove variability. Increasing the compatibility of GoC with standard laboratory equipment and scaling of devices is needed to increase GoC experimental throughput. Further validation of the results obtained through GoCs is required to verify their ability to reliably recapitulate interactions. Advances in the cell biology and microbiology fields ensuring the growth and stability of diverse microbial and human cell types with different growth media and microenvironment requirements will provide essential knowledge to recapitulate physiologically relevant host–microbiota interactions in GoC systems.

## Conclusion

Microfluidic devices are highly useful tools for obtaining a holistic mechanistic understanding of microbiota–host interactions against the background of human genetic, biochemical, and cellular responses in real-time. Nevertheless, the development of such technology is still in its infancy, with challenges still to be overcome. To drive broader adoption of such systems, improved on-chip recapitulation of the intestinal biological and physicochemical microenvironment is needed. Furthermore, advances in cell biology and microbiology are called for organoid-on-a-chip systems for instance to ensure cells’ stability in the presence of whole gut microbiota, as well as preserving a representative gut microbiome composition in GoC systems. Also, monitoring multiple key interdependent interactions between gut communities and the host *in situ* and in real-time remains a major challenge. Further efforts are needed to develop biosensors to profile key activities with high sensitivity, in real-time and in an automated manner.

Like *in vivo* models, current devices are quite diverse, which makes comparison or standardization of models difficult, although some are commercially available. Common standard operating practices must therefore be defined along with a clear regulatory framework. The application of GoC technology as a complementary technology to animal studies, and possibly eventually as a standalone technology will enable personalized drug/diet-host-microbiome preclinical research and translation of patient-specific responses into personalized treatments in clinical practice.
